# The Great Recession and subjective well-being: How did the life satisfaction of people living in the United Kingdom change following the financial crisis?

**DOI:** 10.1371/journal.pone.0201215

**Published:** 2018-08-29

**Authors:** Christopher J. Boyce, Liam Delaney, Alex M. Wood

**Affiliations:** 1 Behavioural Science Centre, Stirling Management School, University of Stirling, Stirling, Scotland; 2 UCD Geary Institute, University College Dublin, Bellfield, Dublin, Ireland; 3 School of Psychological Sciences, University of Manchester, Manchester, England; University of Oxford, UNITED KINGDOM

## Abstract

The financial crisis of 2007/08 precipitated a severe global economic downturn, typically referred to as the Great Recession. However, in the United Kingdom this period has been marked by limited change in national indicators of subjective well-being. We assessed the life satisfaction change in response to the Great Recession in a sample of British adults (*N* = 8,661). We first show that on average the life satisfaction change across the sample was limited. However, average effects may mask substantial amounts of heterogeneity in the data. We therefore explore beyond this average effect to determine whether there were disproportionate changes (losses and gains) in life satisfaction in key sub-groups of the population. We found that individuals experiencing unemployment, who lost income, were sick or disabled, experienced the greatest well-being reductions. Contrastingly the life satisfaction of many individuals did not greatly change following the Great Recession and for some it may have even improved. Our work highlights vulnerable groups that may need additional help during recession periods and also cautions against the over reliance on average measures of well-being.

## Introduction

The financial crisis of 2007/08 precipitated a global economic downturn that due to its relative severity has become known as the Great Recession. In the United Kingdom Gross Domestic Product reached a pre-recession peak in the 1^st^ quarter of 2008 at £422,382 million and fell for the next 5 quarters reaching a recession low of £396,514 million in the 2^nd^ quarter of 2009, an overall drop of 6.1%. Net national income on the other hand fell 11.9% from pre-recessional highs and following a short recovery began a further decline in the final quarter of 2011 [1]. At the same time the unemployment rate rose from 5.2% to a high of 8.5% by October 2011. In 2010, amidst this economic upheaval, a programme to measure national well-being in the UK was launched. The first measures were taken in 2011 and since then the proportion of individuals rating their satisfaction with life as high has risen (e.g., proportion of individuals who responded 9 to 10 on a scale of 0 to 10 where 0 was not at all satisfied and 10 was completely satisfied, see [2]). But how does this compare with pre-recessional levels of well-being and does the reliance on aggregate levels mask what may have been taking place at the individual level? Here we examine how subjective well-being changed in the United Kingdom following the Great Recession and whether effects may have fallen disproportionately on segments of the population.

Recessions are characterized by increases in unemployment and reductions in income. Thus given the abundance of research showing that unemployment has severe long-term negative effects on subjective well-being [3–5] and that income losses have a larger impact on subjective well-being than equivalent gains [6–8] it might be expected that the Great Recession resulted in large and persistent drops in well-being. However, aggregate well-being reductions in the UK and US may have been modest during this period [9]. For example, data from the European Social Survey indicates that life satisfaction and happiness in the UK were relatively stable throughout 2006 to 2010 (life satisfaction on a 0 to 10 scale being 7.13 in 2006, 7.02 in 2008, 7.10 in 2010, and 7.28 in 2012 and happiness on a 0 to 10 scale being 7.43 in 2006, 7.44 in 2008, 7.41 in 2010, and 7.50 in 2012). In the United States average well-being rebounded to pre-recessional levels fairly quickly despite continual economic difficulty [10]. One explanation for these limited effects might be that recessions can sometimes have positive effects. For example, recessions have been linked to improvements in some health indicators [11,12] and it is argued that recessions may increase opportunities to spend more time on otherwise neglected activities, such as home production [13], time with children [13], sleep [14], and exercise [15], that may be of benefit to subjective well-being.

However, another argument as to why there have been limited well-being effects would be that any focus on aggregate statistics may conceal a vast amount of heterogeneity in individual reactions [16]. Since many people were not directly affected by the Great Recession [17] it is possible that large negative subjective well-being effects of the Great Recession were only experienced by a sub-set of the population. If this sub-set were small, then it may have had a very limited effect on overall aggregate levels. Perhaps consistent with this there is evidence that the Great Recession increased the incidence of mental illness in the population [18]. The incidence of mental illness, which is a reflection of difficulties for those at the extreme of the well-being distribution [19], has been shown to have risen mostly among those who lost the most, such as those that became unemployed and the young [20,21].

Here, we estimate the changes in life satisfaction in the population during the main period of economic turbulence. Although our examination is largely exploratory, we make use of the current literature to make several firm predictions as to who is most likely to have been affected by the Great Recession and why. Since increases in unemployment and reductions in income have been demonstrated to have generally negative effects on well-being [3–8] and that national income and unemployment rates fell [1], we first hypothesise that the life satisfaction of the overall population will have on average fallen during this period. However, although some research has shown that individual well-being is somewhat influenced by the circumstances of others [22,23] it is an individual’s own circumstances that matter the most. Thus, if only a relatively small proportion of the population experienced economic difficulty then the overall effect on population life satisfaction may have been small. Our second hypothesis is therefore that any effects from the Great Recession will have fallen disproportionately on those that were directly affected, namely through reduced incomes, increased unemployment, and reduced work hours. An individual experiencing losses in income is less likely to be able fulfil basic needs [24] and also less able to fully participate in society–i.e. through relative consumption practices [22,25] or financing social engagement [26]. Although unemployment, and similarly for lower work hours, may provide more opportunities for social engagement, it is more likely to inhibit their social engagement through feeling their societal value is reduced [27]. Similarly, unemployment and lower work hours may represent an under-utilization of an individual’s skills and therefore result in dissatisfaction [28,29]. It has also been shown that unemployment results in substantial shifts in an individual’s personality [30]. It is also possible that employee participation in decision making at work may have reduced following the Great Recession and this may have similarly reduced satisfaction [31].

We further explore the extent to which well-being over the recession period varied by exposure and hypothesize that the Great Recession will have had some indirect effect on those that are in groups that are the most vulnerable to potential economic shocks. Those that were either unemployed, under-employed, or on a low income before the recession began may have less resources to deal with any economic shock [32]. They may also generally have lower economic security [33] and economic insecurity has been shown to reduce well-being [34]. Thus we examine whether an individual’s employment status influences their well-being over the recession. Similarly, those individuals who, although not directly affected themselves, are part of demographic groups that were directly affected by the Great Recession may also experience economic insecurity due to a greater perceived likelihood of personally experiencing either income loss, unemployment, and/or reductions in work hours. This likely includes the young, those living in certain geographical regions, and those with lower levels of education. We further predict that those groups that are the least vulnerable, namely older people and those more highly educated, are unlikely to have experienced much, if any, well-being falls. In fact perhaps due to relative improvements [22,25] or through the knowledge that their situation isn’t quite as bad as others [35] they may have even experienced increases in their subjective reports of well-being.

We use a large scale nationally representative longitudinal dataset from the United Kingdom to assess both individual and aggregate effects of the Great Recession. We first examine which individuals were more likely to have experienced changes in their income and work hours, and unemployment over the recession period. This allows us to predict the groups that were the most vulnerable and therefore most likely to have experienced reductions in well-being via direct and indirect exposure. We then compare pre-recessional subjective well-being (2006/7) with that during the height of the recession (2009/10) to see who suffered the most.

## Method

### Data

We explore the effects of the Great Recession on well-being by combining data from the British Household Panel Survey (BHPS) and Understanding Society. The BHPS was one of the United Kingdom’s key household panel surveys and was replaced after 18 waves in 2008/2009 by the Understanding Society dataset. Understanding Society began in 2008/2009 and in the first wave it randomly sampled from the British population. However, in the following wave, as well as resampling those in the first wave, Understanding Society also incorporated the original BHPS sample members. Although there was some attrition between datasets that was higher than between each wave of the BHPS the two datasets can be combined such that we can track what happened to individual well-being following the Great Recession. Since the financial crisis that precipitated the Great Recession took place in 2007/08 we use data from 2006/07 (BHPS wave 16) as the pre-recessional period and data from 2009/10 (Understanding Society wave 2) as our recessional period. Although it is possible to use different periods 2006/07 represents a period before there was any knowledge of a global economic crisis and 2009/10 represents a period at the height of the Great Recession yet before periods of austerity which may confound our results. In our analyses we included all individuals (*N* = 8,661) that answered a question of life satisfaction question in both waves.

### Empirical strategy

Our empirical strategy to establish how subjective well-being (SWB) changed as a result of the Great Recession is depicted in Equation 1.

$$SWB_{T}=\beta_{1}SWB_{T-1}+\beta_{2}Individual\;recession\;exposure\;characteristics+\beta_{3}Pre-recession\;characteristics\;(demographic,\;socio-economic)+\varepsilon$$

Here, we predict SWB during the recession at T from pre-recessional SWB at T-1 such that we estimate residualised changes in well-being to avoid issues with regression to the mean [36]. We designed the specifications to include key demographic variables that are standard in both well-being and financial behaviour analysis. We further added variables driven by previous research on the impact of recessions. We did not iterate on the specification after producing the initial findings but we did reduce the number of variables at the revision stage to simplify the paper and reduce reviewer concerns about multi-collinearity. To determine the factors that are the most important contributors to change in well-being over this period we include recession and pre-recession characteristics in our regression. Our individual recession exposure characteristics include changes in income and changes in work hours, and whether any unemployment was experienced. Our pre-recession characteristics, as detailed more below, include demographics (age, gender, education, and geographical location) and socio-economic circumstances (employment status, number of hours worked, household income, household size) before the recession period.

Our choice to use a residualised change model, as opposed to a fixed effect model, is that it enables both change and level variables to be analysed. A fixed effect model only focuses on the within-person variation and ignores all between-person information [37] and this has been shown to be important for well-being research [38]. Thus the use of a fixed effect model here would not enable us to examine the extent to which pre-recessional level exposure effects were important.

### Measures

#### Subjective well-being

Subjective well-being data has long been argued to be a useful proxy for utility, and there is good evidence to validate this claim [39–41]. The analysis of subjective well-being has therefore become increasingly accepted as a viable tool in economic analysis [42]. Here, we operationalize well-being using a life satisfaction measure contained in both the BHPS and Understanding Society. Life satisfaction is an evaluative well-being measures and has been reliably used throughout economic well-being research [43]. We also assessed changes in well-being using the 12-item General Health Questionnaire [44,45]. Results were qualitatively similar and in the interests of parsimony the results are not reported.

*Life satisfaction* is measured here using a one-item scale. Participants responded to the question “*how dissatisfied or satisfied are you with your life overall*?” on a 7-point scale, from 1 (not satisfied at all) to 7 (completely satisfied). Hence, higher scores represent greater satisfaction with life.

**Fig 1:**
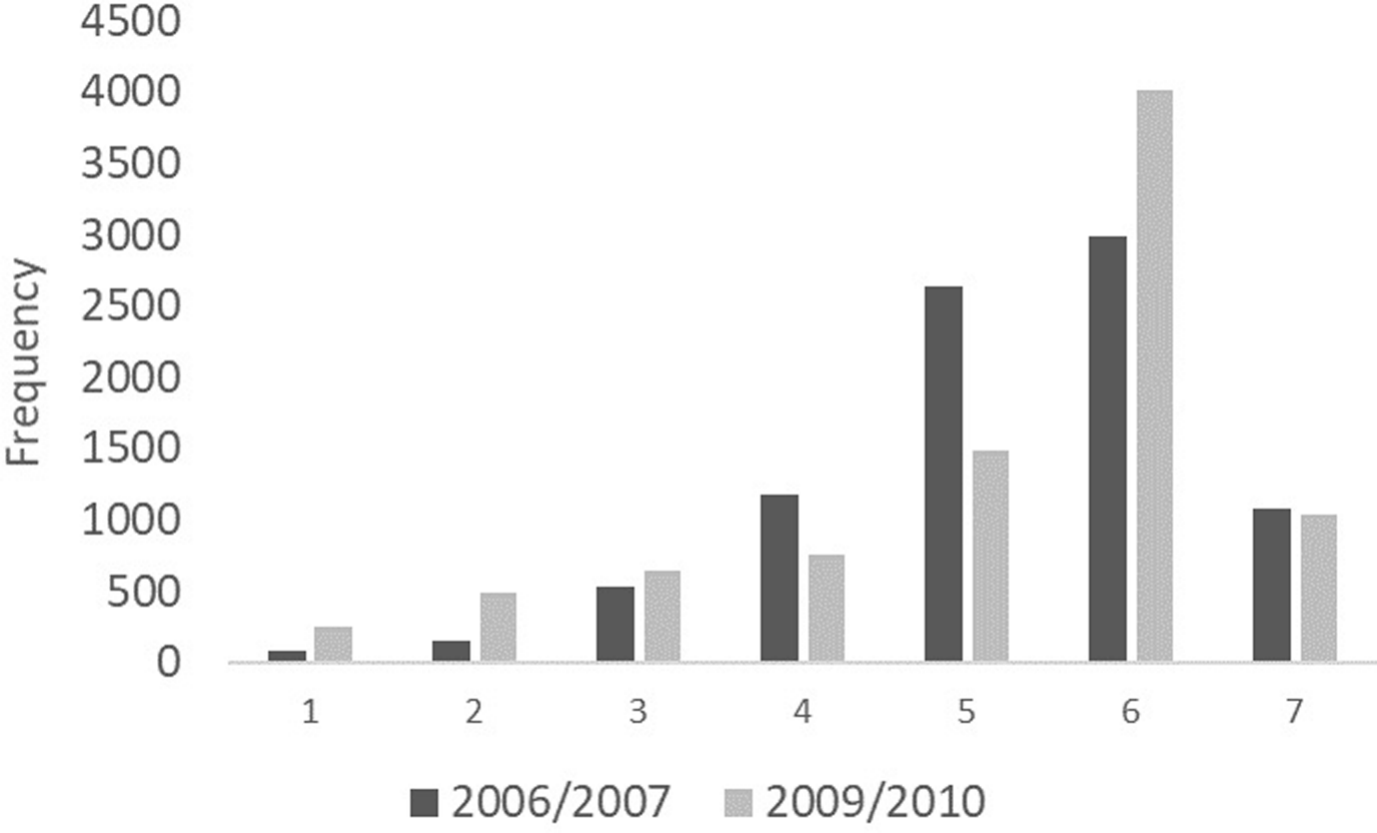
A histogram of life satisfaction in 2006/07 and 2009/10.

Table 1 provides descriptive statistics for our sample. In 2006/07 mean life satisfaction was 5.24 (*SD =* 1.22), whereas is 2009/10 it was 5.18 (*SD =* 1.50) and this difference was statistically significant (*t =* 3.44, *p <* 0.01). Fig 1 provides a histogram of life satisfaction across 2006/07 and 2009/10. The mean values represent the scores for individuals that answered each of the well-being measures in both time periods, whereas the mean values in 2007/08 & 2008/09 includes everyone that answered the well-being measures in 2006/07 and 2009/10 and that specific time-period. On average well-being seems to have shown some worsening. Although the differences between 2006/07 and 2009/10 are significant the magnitude of the effect is small, representing in both cases 0.05 of a standard deviation. However, for both well-being indictors we observe that there are changes in standard deviations and skewness. The changes in standard deviations and skewness suggest that scores have not only become more spread out but that there is greater skew towards the lower end of the well-being distribution. This suggests that focusing on average well-being effects may conceal that many of the negative effects of the Great Recession were concentrated among sub-sets of the population.

**Table 1:** Descriptive statistics and baselines changes in life satisfaction by group (*N* = 8,661).

Variable	Mean	Standard Deviation	Change in life satisfaction by group	Proportion of observations missing (%)
Life satisfaction in 2009/2010	5.18	1.50		0
Life satisfaction in 2006/2007	5.24	1.22		0
Age	46.40	17.34		0.01
Male	0.44	0.50	-0.07	
Female	0.56	0.50	-0.05	0
Education				
No qualifications	0.25	0.43	-0.16**	0
Degree	0.16	0.37	0.07*	0
Other higher degree	0.07	0.26	0.04	0
A-levels etc.	0.19	0.40	-0.03	0
O-levels etc.	0.26	0.44	-0.05	0
Other qualification	0.05	0.21	-0.16*	0
Missing Education	0.02	0.13	-0.25†	0
Region				
London	0.04	0.20	-0.14†	0
The North	0.16	0.36	-0.03	0
Midlands and the East	0.13	0.33	0.02	0
The South	0.04	0.20	-0.02	0
Wales	0.17	0.38	-0.02	0
Scotland	0.18	0.38	-0.03	0
Northern Ireland	0.15	0.36	-0.24**	0
Region missing	0.01	0.04	-0.08	0
**Individual recession exposure characteristics**				
Experienced some unemployment	0.09	0.29	-0.12†	0.24
Change in hours worked	-0.87	13.33		0.22
Log household income change	0.18	0.73		1.64
**Pre-recession socio-economic characteristics T-1**				
Employment Status				
Employed	0.51	0.50	0.00	0
Self-employed	0.07	0.26	-0.13*	0
Unemployed	0.03	0.17	0.09	0
Retired	0.20	0.40	-0.13**	0
On maternity leave	0.01	0.07	-0.53†	0
Looking after family	0.07	0.25	-0.09	0
Full-time student	0.04	0.20	-0.27**	0
Long-term sick/disabled	0.04	0.20	-0.03	0
Government training scheme	0.00	0.04	-0.5	0
Doing something else	0.01	0.09	0.11	0
Job status missing/unknown	0.02	0.15	-0.25*	0
Household income (monthly)	2,822	1,896		0
Hours worked	17.82	18.31		0.09
Household size	2.85	1.36		0

Table 1 also provides the baselines changes in life satisfaction by group. We see from the simple descriptive statistics and education and pre-recession employment status seem to play an important role. In our later regression analysis, we standardize our life satisfaction variable with a mean of zero and a standard deviation of one. This standardization enables our effects to be meaningfully interpreted with respect to standard deviation changes.

#### Pre-recession and individual recession exposure characteristics

We are interested in a number of factors that might explain which individuals experienced the largest reductions in their well-being following the recession. These can be grouped into individual recession exposure characteristics, pre-recession demographic characteristics, and pre-recession socio-economic characteristics. *Individual recession exposure characteristics* were the key predictor variables and represented changes in individual circumstances that may have occurred directly as a result of the recession between 2006/07 and 2009/10. This included whether an individual experienced some unemployment (at any time between 2006/07 and 2009/10, *n =* 800), changes in their work hours (from 2006/07 to 2009/10), or changes in their household’s log-linear income (from 2006/07 to 2009/10). *Pre-recession demographic characteristics* recorded in 2006/07 included the individual’s age, their sex (male, female), their education level (degree, other higher degree, A-levels and equivalents, O-levels and equivalents, other qualifications, no qualifications), and the region of the United Kingdom in which they live (the North, Midlands and the East, the South, London, Wales, Scotland, Northern Ireland). *Pre-recession socio-economic characteristics* recorded in 2006/07 included employment status (self-employed, employed, retired, on maternity leave, looking after family, full-time student, long term sick/disabled, Government training scheme, doing something else), their household’s log-linear income, hours worked, and household size in which they lived (log-linearized).

#### Missing data

Of the full sample (*N* = 8,661) that had scores for life satisfaction in 2006/07 and 2009/10 we observed a small amount of missing data. For many of the categorical variables (education level, region, employment status) where there was missing data we included an extra category to indicate those individuals who had missing data for that variable. Where there were missing values for the binary and continuous variables; whether they experienced some unemployment (0.2%), changes in their work hours (0.2%), or changes in their household’s log-linear income (1.6%), hours worked (0.1%), we imputed values using multiple imputation [46]. Unless these items are missing completely at random (MCAR), list-wise deletion, or imputing sample wide or item averages have been shown to lead to biased estimates [47]. Multiple imputation imputes a series of missing values based on estimates from other observed variables and more appropriately accounts for the statistical uncertainty in the imputations than many other commonly used techniques [47]. We specifically used multiple imputation chained equations (MICE; [48]), which is a technique whereby for each of the multiple imputations a series of sequential regressions appropriate to the missing variable (e.g., predictive mean matching for the continuous variables, logistical regressions for binary variables, and ordered logistical regression for the categorical variable) are carried out in an iterative fashion. We obtained 5 imputations (based on 10 sequential iterations using MICE) and we pooled each of our imputations to produce our final estimates. Overall the approach we took to missing data resulted in an additional 179 (0.2%) observations which would have otherwise been excluded from our analysis. Given the amount of missing data overall our chosen number of 5 imputations provided a relative efficiency of 97.5%, where >95% is an acceptable level (see [49]). The results were not substantively affected by the inclusion of imputed data, although some of the coefficients were estimated with more or less precision. We carried out all our analyses using Stata 12 [50].

## Results

We expect to find that the largest well-being effects of the Great Recession were experienced by those who had the largest economic difficulty. However, we also expect that those, though not necessarily directly affected, that were in demographic groups more likely to be affected would experience well-being drops due to increased uncertainty. Thus we begin our analysis by first exploring which groups of people were more likely to experience economic difficulty in the form of income reductions, loss of work hours, and unemployment. Table 2 investigates which demographic groups experienced the largest changes in log income, changes in work hours, and experienced some unemployment over the period from 2006/07 to 2009/10. From Model 1 in Table 2 we observe that on average over this time period there were rises in log household income. We see however that those living outside of London experienced higher increases in income. With respect to age it is those that are older that are more likely to have experienced lower income increases, whilst the young experienced higher income increases. Level of education did not influence the likelihood of experiencing an income change. However, when we examine the relevance of education for unemployment and changes in work hours in Models 2 and 3 respectively there was an important role for education. Those with low levels of education were more likely to experience unemployment and a reduction in their working hours. The young members of the sample were more likely to be unemployed yet more likely to see their hours of work increase relative to those that were older. Although we cannot be sure why incomes and work hours reduced (for example, transitions to the workplace and retirement may have been a key factor) overall Table 2 suggests that the greatest economic difficulty occurred to those that were young and those with lower levels of education. Those highly educated or those that were older being less likely to experience economic difficulty. Thus once we control for the economic difficulty an individual experienced we may still expect to see decreased well-being among the young and those with lower levels of education following the Great Recession due to increased uncertainty.

**Table 2:** Who experienced income and work hour changes, and/or job loss during the Great Recession? Differences relative to uneducated men aged between 45 and 50 and living in London.

	(1)	(2)	(3)
	From 2006/7 to 2009/10		
VARIABLES	Change in log household income	Unemployment	Work hours
**Pre-recession demographic characteristics T-1**			
Age	-0.009**	-0.011**	-0.586**
	(0.003)	(0.001)	(0.048)
Age-Squared/1000	0.055*	0.068**	5.080**
	(0.026)	(0.010)	(0.439)
Female	0.011	-0.037**	0.836**
	(0.016)	(0.006)	(0.296)
Excluded dummy: No qualifications			
Degree	0.045*	-0.086**	-0.126
	(0.023)	(0.010)	(0.461)
Other higher degree	-0.002	-0.082**	-0.732
	(0.028)	(0.010)	(0.546)
A-levels etc.	0.010	-0.057**	-0.643
	(0.026)	(0.010)	(0.455)
O-levels etc.	-0.030	-0.036**	-0.712
	(0.023)	(0.009)	(0.399)
Other qualification	0.005	0.021	-2.408**
	(0.039)	(0.020)	(0.844)
Missing Education	0.133	-0.003	-1.684
	(0.110)	(0.030)	(1.352)
Excluded regional dummy: London			
The North	-0.097*	0.005	-0.605
	(0.044)	(0.017)	(0.753)
Midlands and the East	-0.166**	0.005	-0.335
	(0.045)	(0.017)	(0.782)
The South	-0.155**	-0.030†	0.474
	(0.043)	(0.016)	(0.758)
Wales	-0.112**	0.002	-0.640
	(0.043)	(0.016)	(0.745)
Scotland	-0.066	-0.005	-0.573
	(0.044)	(0.016)	(0.746)
Northern Ireland	-0.183**	-0.020	-0.338
	(0.044)	(0.016)	(0.757)
Region missing	-0.272*	-0.017	-6.725
	(0.134)	(0.082)	(4.075)
Constant	0.598**	0.498**	14.388**
	(0.083)	(0.033)	(1.431)
Adjusted R-Squared	.014	.082	.030
Observations	8661	8661	8661

Robust standard errors in parentheses

** p<0.01,

* p<0.05,

† p<0.1

Table 3 shows the residualised changes in life satisfaction from 2006/7 to 2009/10. A model with no controls suggests that life satisfaction fell on average by 0.02 standard deviations (*p* < .05). A comparison of this to the baseline difference in Table 1 illustrates the importance of controlling for baselines levels using a residualised change model. Model 1 shows that changes in life satisfaction depend upon demographic factors. For example, an uneducated man of average age (46.4) living in London experienced on average a decrease in life satisfaction of 0.16 standard deviations. However, those that were older and educated were somewhat protected from well-being decreases and may have experienced increases in their well-being. The quadratic in age predicts a u-shape relationship with life satisfaction and suggests that those who were approximately 33 experienced the largest reductions in life satisfaction (-0.08 *SD*) and that at around 63 the effect turns positive. Table 1 suggests that it was those that younger and less educated who were more likely to experience decreases in income and the number of hours worked, and some unemployment over the recession period and the result that the life satisfaction of the least educated and younger individuals is most affected is generally consistent. However, if it were just the direct effects that were important we would expect that once individual recession exposure characteristics are controlled for then these demographic factors may no longer be individually significant.

**Table 3:** Subjective well-being changes from 2006/7 to 2009/2010 (individual recession exposure characteristics, pre-recession demographic and socio-economic variables): Differences relative to an uneducated average aged man, living in in London, and who remained employed.

	(1)	(2)
VARIABLES	Life satisfaction	Life satisfaction
Lag of SWB variable	0.459**	0.427**
	(0.014)	(0.014)
**Pre-recession demographic characteristics T-1**		
Age	-0.005	-0.007
	(0.004)	(0.004)
Age-Squared/1000	0.078*	0.081†
	(0.037)	(0.046)
Female	0.012	0.008
	(0.022)	(0.024)
Excluded dummy: No qualifications		
Degree	0.237**	0.131**
	(0.038)	(0.040)
Other higher degree	0.213**	0.135**
	(0.045)	(0.045)
A-levels etc.	0.108**	0.044
	(0.038)	(0.038)
O-levels etc.	0.118**	0.075*
	(0.035)	(0.035)
Other qualification	-0.016	-0.034
	(0.062)	(0.061)
Missing Education	0.018	-0.017
	(0.097)	(0.100)
Excluded regional dummy: London		
The North	0.048	0.074
	(0.060)	(0.060)
Midlands and the East	0.122*	0.150*
	(0.061)	(0.061)
The South	0.077	-0.083
	(0.059)	(0.059)
Wales	0.087	0.121
	(0.060)	(0.060)
Scotland	0.075	0.109†
	(0.060)	(0.059)
Northern Ireland	-0.022	0.028
	(0.061)	(0.062)
Region missing	0.185	0.198
	(0.177)	(0.172)
**Individual recession exposure characteristics**		
Experienced some unemployment		-0.173**
		(0.047)
Change in hours worked		0.000
		(0.001)
Log household income change		0.072**
		(0.022)
**Pre-recession socio-economic characteristics T-1**		
Excluded dummy: Employed		
Self-employed		-0.100†
		(0.059)
Unemployed		-0.057
		(0.082)
Retired		-0.031
		(0.058)
On maternity leave		-0.243
		(0.179)
Looking after family		-0.122*
		(0.062)
Full-time student		-0.116
		(0.073)
Long-term sick/disabled		-0.531**
		(0.076)
Government training scheme		-0.472†
		(0.284)
Doing something else		-0.111
		(0.146)
	(1)	(2)
VARIABLES	Life satisfaction	Life satisfaction
Job status missing/unknown		-0.052
		(0.091)
Log household income		0.085*
		(0.024)
Hours worked		-0.001
		(0.001)
Log of household size		-0.063*
		(0.029)
Constant	-0.164†	0.029
	(0.098)	(0.120)
R-Squared	.151	.162
Observations	8,661	8,661

Robust standard errors in parentheses

** p<0.01,

* p<0.05,

† p<0.1

Model 2 Table 3 therefore further includes individual recession exposure characteristics, as well as pre-recession socio-economic characteristics. The baseline comparison group in these regressions is an unmarried uneducated man of average age, living in London, and who remained employed throughout the period. From the constant term in the regression the data suggests that this group experienced life satisfaction reductions of 0.03 standard deviations. Although indicating higher life satisfaction these effects are not significant. However, as predicted we observe that experiencing unemployment and losses in income were associated with reductions in well-being. For example, an individual experiencing some unemployment experienced losses in life satisfaction of 0.17 standard deviations compared with those not experiencing unemployment over this period. Unemployment is typically accompanied by an income loss and with each standard deviation loss in income there is an associated 0.07 further standard deviation decrease in life satisfaction. In addition, we find that those that were sick or disabled in 2006/07 experienced large falls in their life satisfaction over this time period (0.53 standard deviations). A high household income prior to the recession also offered some protection for individuals. The inclusion of the individual exposure and pre-recessional socio-economic characteristics accounts for some of the previous demographic differences from Model 1. However, we still see education is an important predictors of adverse life satisfaction responses to the Great Recession. Thus the baseline education effect is likely to have been largely driven by other correlated factors.

**Fig 2:**
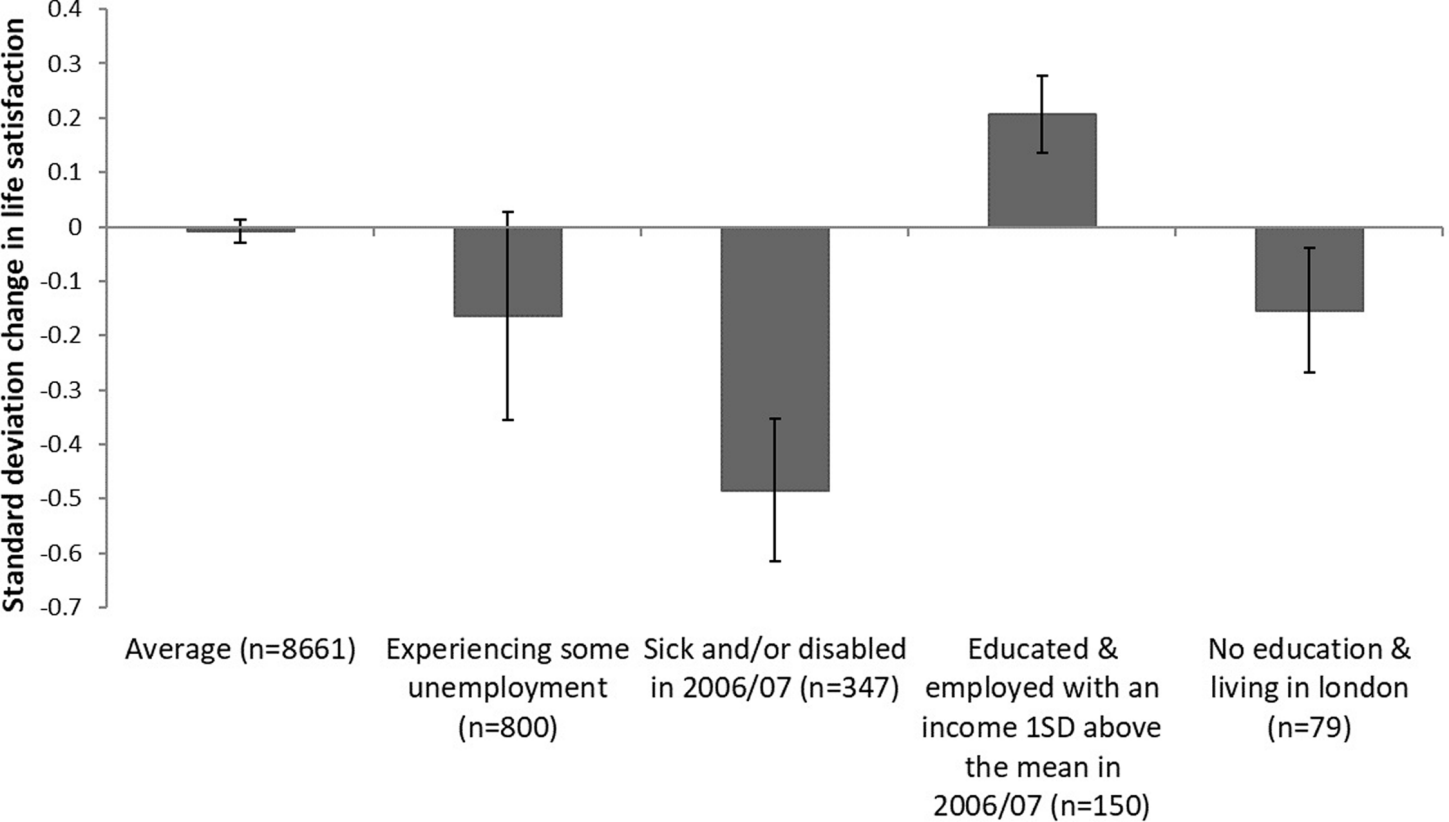
Changes in life satisfaction following the Great Recession for different identifiable groups of individuals.

Our results are displayed graphically in Fig 2, which shows the regression-adjusted standard deviation changes in life satisfaction for selected groups of the population. As can be seen, declines in life satisfaction were particularly pronounced for those entering the Great Recession in a state of sickness or disability. The experience of unemployment also strongly predicted declines in life satisfaction. We further see that those who are highly educated and that were highly educated and in employment and receiving an income at least 1 standard deviation above mean levels experienced an increase in their life satisfaction. Thus this highlights that any focus on only average changes in well-being may mask that whilst some individuals may have registered little change in their well-being, or even improved, a sub-group is likely to have experienced much larger changes.

## Discussion

We explored how subjective well-being in the United Kingdom changed following the Great Recession. We found, as hypothesised, that on average there were only small changes in national well-being but that behind the average there were large changes that were concentrated among key sub-groups of the population. This primarily included those that experienced some unemployment or lost income during the recession period. We also found that those who were sick or disabled prior to the recession were also more acutely affected. The well-being of many individuals did not change following the Great Recession and may have even improved. This included individuals who were older, had higher incomes, and were more highly educated. Our study is one of the first to document subjective well-being before and after the Great Recession and has important implications for the use of well-being indicators to guide policy [51,52].

Our research highlights that concentrating only on average effects across a population can result in misleading conclusions as to how individuals’ well-being might have been affected. Often average effects conceal that there is a huge diversity in how people might respond. For example, the well-being effects from changes in income can vary across individuals [53] and this may depend on a variety of factors, such as an individual’s age [54], position in the well-being distribution [55,56], and whether the income change is a loss or a gain [7]. These differences can be such that a life experience may have little or no effect on their well-being whilst for others the effect is more severe [57]. Thus any observation of only limited change in national indicators of well-being, as indicated for example by recent UK indicators of well-being [2], may hide that some individuals have experienced large reductions in well-being whilst others have experienced increases. In spite of one of the largest economic recessions of the modern period our research highlights that by focusing on averages one might conclude that there has been limited well-being effects. Our results indicate that economic fluctuations may have particularly pronounced effects on identifiable groups that represent only a small overall percentage of the population. Thus a headline figure comparing well-being from year-to-year can miss large changes in the welfare of specific groups, such as those with particular types of disability. It is not optimal to use a metric with such potential for misleading conclusions without a high degree of supplementary analysis. It has been documented that increases in suicide rates during the recession period have arisen from at-risk groups [58], and further research is needed to reconcile well-being analyses and epidemiological analyses of suicide and attempted suicide.

With regard to specific policy conclusions our results suggest that certain groups may need additional support in times of crisis to mitigate potential reductions in well-being. It is those that were already more vulnerable, for example those who were sick or disabled, or unemployed, that experienced the largest well-being drops following the Great Recession. Furthermore, the fiscal policy response in many countries has involved austerity measures that have fallen more heavily on groups that our research highlights as experiencing the largest well-being drops. Thus these groups may have therefore been doubly affected as a result of the Great Recession [20].

One reason for our limited overall effect in the population may be due to the time-period analysed. Although our baseline measures taken in 2006/07 represented a period before there was any knowledge of a global financial crisis and the observation in 2009/10 was near to the height of the Great Recession this may not have been the optimal period of analysis. Indeed, any well-being effects may have been more pronounced at an earlier time-period during the Great Recession. On this issue we were constrained by the available data as there was no data available for this group of individuals in 2008/09 in Understanding Society. Similarly, it is possible that the effects of the Great Recession may have taken longer to materialise with a later time period being optimal. However, in 2010 the United Kingdom embarked on a period of austerity and thus it would have been difficult to separate any recession effects from the effects of austerity [59,60]. This hampered our ability to be able to assess any meaningful counterfactuals and limits our ability to identify any clear causal effects. Ideally we would have liked higher frequency data and assessed the trajectory of various groups across several data points. There are also likely selection effects. 79.4% of respondents in the final wave of the British Household Panel Survey were successfully re-interviewed in the Understanding Society [61]. Thus certain groups of the population are likely to be under-represented and it is possible that the likelihood of attrition is linked to our subjective well-being variables. For example, those experiencing unemployment may be more likely to attrition from the dataset, and this may itself be a function of the well-being influence of the unemployment experience. There is also the possibility that multi-collinearity may have limited our ability to detect effects across some variables. There were a number of statistical differences that were present in the descriptive statistics but not the regression analysis (see Table 1). A further limitation is accounting for time-invariant characteristics. Our analyses used an Ordinary Least Squares estimator. Although we assessed residualised changes and controlled for a number of potentially correlated factors, which included those identified in the economic literature [43], it is still likely that time-invariant characteristics may have explained some of our results. A fixed effect model is typically considered to be a better model to handle time-invariant characteristics [62] but since many of our key variables were measured at only one time point (for example our pre-recessional characteristics) this estimator would have been inappropriate. However, the inclusion of personality variables, sometimes considered a time-invariant variable [63], did not change our results.

Our research contributes to the discussion around the effects of recessions more generally. Recessions have been shown to sometimes have positive effects on health [12] and our research highlights that for many individuals there are likely to also be positive well-being effects. It has been shown that economic growth is associated with little, if any, increases in well-being [8,64–66] and one reason for this may be due to individuals having less opportunity to find value in other things in life that contribute to their well-being, such as home production [13], time with children [13], sleep [14], and exercise [15]. Our research supports the notion that for many people economic growth or recession has limited effects on well-being but, for an unlucky minority, recessions can hurt and from a well-being perspective such individuals need greater support.

## Supporting information

S1 File Stata do file PLOS R1.pdf. Stata do file.
(PDF)
