# Supplementary material for: The Great Recession and subjective well-being: How did the life satisfaction of people living in the United Kingdom change following the financial crisis?
Source: PLoS One. 2018 Aug 29;13(8):e0201215. doi: 10.1371/journal.pone.0201215 (PMC6114278; doi:10.1371/journal.pone.0201215)
Supplement: S1 File — Stata do file. (PDF) [file pone.0201215.s001.pdf]

Copy of the Stata .do file for "The Great Recession and subjective well-being: How did the subjective well-being of people living in the United Kingdom change following the financial crisis?"

Data comes from the UK Data Service (ESRC data archive). Access requires registration.

DOI's for datasets used:
British Household Panel Survey - UKDA-5151
http://doi.org/10.5255/UKDA-SN-5151-1
Understanding Society - UKDA-6614
http://doi.org/10.5255/UKDA-SN-6849-10

Step 1: Merging original datasets and obtaining relevant variables

```
use "C:\UKDA-5151-stata8\stata8\xwaveid.dta", clear
rename doby yearofbirth
rename dobm monthofbirth
keep pid phid qhid rhid sex monthofbirth yearofbirth
***////// wave p //////***
merge m:1 phid using "C:\UKDA-5151-stata8\stata8\phhresp.dta"
rename phid hid16
rename phsval hsevalue16
rename phscost hsecost16
rename pmgold mortgageamt16
rename pxpmg monthlymortloan16
rename pnkids children16
rename pfihhyr hhincome16
rename phhsize hhsize16
rename pxphsdf hsepayprobs16
rename pxphsd1 hsepayborrow16
rename pxphsd2 hsepaycutbacks16
rename pxphsdb hsepaybehind16
rename pxphp loans16
rename pxphpdf loanburden16

keep pid-rhid hid16 hsevalue16 hsecost16 loanburden16 loans16 mortgageamt16
monthlymortloan16 hsepayprobs16 hsepayborrow16 hsepaycutbacks16 hsepaybehind16
hhincome16 children16 hhsize16

merge 1:1 pid using "C:\UKDA-5151-stata8\stata8\pindresp.dta"

rename pfisit finsituation16
rename pfisitc finsituationchange16
rename psave saves16
rename psaved savesamt16
rename psavreg saveregular16
rename psavlt longtermsaver16
rename pfiyri ivtincome16
rename pdoid dayofint16
rename pdoim monthofint16
```

```
rename pjbsic92 industry92_16
rename psmoker smoker16
rename pncigs cigssmoked16
rename pjbsoc occupation90_16
rename pjbsemp sfemp16
rename phoh hoh16
rename page age16
rename pjshrs hrswrksemp16
rename pjbttwt commutehrs16
rename pregion region16
rename ptenure hsetenancy16
rename pqfachi education16
rename pmlstat maritalstatus16
rename phldsbl1 disable16
rename pfiyr income16
rename pjbstatt jobstatus16
rename pjbhrs hrswrk16
rename pjbot overtime16
rename phlghq1 mental36hlth16
rename phlghq2 mental12hlth16
rename pjbsat jobsat16
rename pjbsat2 paysat16
rename pjbsat4 jobsecuritysat16
rename pjbsat7 jobhrssat16
rename phlstat subjhlth16
rename plfsat1 hlthsat16
rename plfsat2 hhincsat16
rename plfsat3 flatsat16
rename plfsat4 partnersat16
rename plfsat5 job2sat16
rename plfsat6 socialsat16
rename plfsat7 leistimesat16
rename plfsat8 leisusesat16
rename plfsato lifesat16
rename plfsatl lifelysat16
rename pfrna neighbourchat16
rename pfrnb meetingpeople16
rename pfrnc spokeninlastwk16
rename placta wlkswimsprt16
rename plactb livesprt16
rename plactc cinema16
rename plactd theatre16
rename placte eatout16
rename plactf drinkout16
rename placth garden16
rename placti diycar16
rename plactj eveclass16
rename plactk attendgrps16
rename plactl volunteer16
rename pvote4 politicalparty16
```

rename pjbstatl jobstatusly16
rename pnjuwks unemployedwks16
rename pnjusp unemployedspells16
rename pnjbsp employedspells16
rename pnjbnew employeddiffemployers16

keep pid-hhincome16 employeddiffemployers16 employedspells16 unemployedspells16 unemployedwks16 jobstatusly16 politicalparty16 dayofint16 longtermsaver16 saveregular16 monthofint16 saves16 savesamt16 ivtincome16 finsituation16 finsituationchange16 smoker16 cigssmoked16 hoh16 age16 region16 hsetenancy16 education16 maritalstatus16 disable16 jobstatus16 income16 hrswrk16 overtime16 commutehrs16 industry92_16 occupation90_16 sfemp16 hrswrksemp16 mental36hlth16 mental12hlth16 jobsat16 paysat16 jobsecuritysat16 jobhrssat16 subjhlth16 hlthsat16 hhincsat16 flatsat16 partnersat16 job2sat16 socialsat16 leistimesat16 leisusesat16 lifesat16 lifelysat16 neighbourchat16 meetingpeople16 spokeninlastwk16 wlkswimsprt16 livesprt16 cinema16 theatre16 eatout16 drinkout16 garden16 diycar16 eveclass16 attendgrps16 volunteer16

////// wave q //////

merge m:1 qhid using "C:\UKDA-5151-stata8\stata8\qhhresp.dta"

rename qhid hid17
rename qhsval hsevalue17
rename qhscost hsecost17
rename qmgold mortgageamt17
rename qxpmg monthlymortloan17
rename qnkids children17
rename qfihhyr hhincome17
rename qhhsize hhsize17
rename qxphsdf hsepayprobs17
rename qxphsd1 hsepayborrow17
rename qxphsd2 hsepaycutbacks17
rename qxphsdb hsepaybehind17
rename qxphp loans17
rename qxphpdf loanburden17

keep pid-mental12hlth16 hid17 hsevalue17 hsecost17 loanburden17 loans17 mortgageamt17 monthlymortloan17 hsepayprobs17 hsepayborrow17 hsepaycutbacks17 hsepaybehind17 hhincome17 children17 hhsize17

merge 1:1 pid using "C:\UKDA-5151-stata8\stata8\qindresp.dta"

rename qfisit finsituation17
rename qfisitc finsituationchange17
rename qsave saves17
rename qsaved savesamt17
rename qsavreg saveregular17
rename qsavlt longtermsaver17
rename qfiyri ivtincome17
rename qdoid dayofint17

```
rename qdoim monthofint17
rename qjbsic92 industry92_17
rename qsmoker smoker17
rename qncigs cigssmoked17
rename qjbsoc occupation90_17
rename qjbsemp sfemp17
rename qhoh hoh17
rename qage age17
rename qjshrs hrswrksemp17
rename qjbttwt commutehrs17
rename qregion region17
rename qtenure hsetenancy17
rename qqfachi education17
rename qmlstat maritalstatus17
rename qhldsbl1 disable17
rename qfiyr income17
rename qjbstatt jobstatus17
rename qjbhrs hrswrk17
rename qjbot overtime17
rename qhlghq1 mental36hlth17
rename qhlghq2 mental12hlth17
rename qjbsat jobsat17
rename qjbsat2 paysat17
rename qjbsat4 jobsecuritysat17
rename qjbsat7 jobhrssat17
rename qhlstat subjhlth17
rename qlfsat1 hlthsat17
rename qlfsat2 hhincsat17
rename qlfsat3 flatsat17
rename qlfsat4 partnersat17
rename qlfsat5 job2sat17
rename qlfsat6 socialsat17
rename qlfsat7 leistimesat17
rename qlfsat8 leisusesat17
rename qlfsato lifesat17
rename qlfsatl lifelysat17
rename qfrna neighbourchat17
rename qfrnb meetingpeople17
rename qfrnc spokeninlastwk17
rename qvote4 politicalparty17
rename qjbstatl jobstatusly17
rename qnjuwks unemployedwks17
rename qnjusp unemployedspells17
rename qnjbsp employedspells17
rename qnjbnew employeddiffemployers17

keep pid-hhincome17 employeddiffemployers17 employedspells17 unemployedspells17 unemployedwks17 jobstatusly17 politicalparty17 dayofint17 longtermsaver17 saveregular17 monthofint17 saves17 savesamt17 ivtincome17 finsituation17 finsituationchange17 smoker17 cigssmoked17 hoh17 age17 region17 hsetenancy17 education17 maritalstatus17
```

disable17 jobstatus17 income17 hrswrk17 overtime17 commutehrs17 industry92_17 occupation90_17 sfemp17 hrswrksemp17 mental36hlth17 mental12hlth17 jobsat17 paysat17 jobsecuritysat17 jobhrssat17 subjhlth17 hlthsat17 hhincsat17 flatsat17 partnersat17 job2sat17 socialsat17 leistimesat17 leisusesat17 lifesat17 lifelysat17 neighbourchat17 meetingpeople17 spokeninlastwk17

////// wave r //////

merge m:1 rhid using "C:\UKDA-5151-stata8\stata8\rhhresp.dta"

rename rhid hid18
rename rhsval hsevalue18
rename rhscost hsecost18
rename rmgold mortgageamt18
rename rxpmg monthlymortloan18
rename rnkids children18
rename rfihhyr hhincome18
rename rhhsize hhsize18
rename rxphsdf hsepayprobs18
rename rxphsd1 hsepayborrow18
rename rxphsd2 hsepaycutbacks18
rename rxphsdb hsepaybehind18
rename rxphp loans18
rename rxphpdf loanburden18

keep pid-mental12hlth17 hid18 hsevalue18 hsecost18 loanburden18 loans18 mortgageamt18 monthlymortloan18 hsepayprobs18 hsepayborrow18 hsepaycutbacks18 hsepaybehind18 hhincome18 children18 hhsize18

merge 1:1 pid using "C:\UKDA-5151-stata8\stata8\rindresp.dta"

rename rfisit finsituation18
rename rfisitc finsituationchange18
rename rsave saves18
rename rsaved savesamt18
rename rsavreg saveregular18
rename rsavlt longtermsaver18
rename rfiyri ivtincome18
rename rdoid dayofint18
rename rdoim monthofint18
rename rjbsic92 industry92_18
rename rsmoker smoker18
rename rncigs cigssmoked18
rename rjbsoc occupation90_18
rename rjbsemp sfemp18
rename rhoh hoh18
rename rage age18
rename rjshrs hrswrksemp18
rename rjbttwt commutehrs18
rename rregion region18

```
rename rtenure hsetenancy18
rename rqfachi education18
rename rmlstat maritalstatus18
rename rhldsbl1 disable18
rename rfiyr income18
rename rjbstatt jobstatus18
rename rjbhrs hrswrk18
rename rjbot overtime18
rename rhlghq1 mental36hlth18
rename rhlghq2 mental12hlth18
rename rjbsat jobsat18
rename rjbsat2 paysat18
rename rjbsat4 jobsecuritysat18
rename rjbsat7 jobhrssat18
rename rhlstat subjhlth18
rename rlfsat1 hlthsat18
rename rlfsat2 hhincsat18
rename rlfsat3 flatsat18
rename rlfsat4 partnersat18
rename rlfsat5 job2sat18
rename rlfsat6 socialsat18
rename rlfsat7 leistimesat18
rename rlfsat8 leisusesat18
rename rlfsato lifesat18
rename rlfsatl lifelysat18
rename rfrna neighbourchat18
rename rfrnb meetingpeople18
rename rfrnc spokeninlastwk18
rename rlacta wlkswimsprt18
rename rlactb livesprt18
rename rlactc cinema18
rename rlactd theatre18
rename rlacte eatout18
rename rlactf drinkout18
rename rlacth garden18
rename rlacti diycar18
rename rlactj eveclass18
rename rlactk attendgrps18
rename rlactl volunteer18
rename roprlg1 religion18
rename rvote4 politicalparty18
rename rjbstatl jobstatusly18
rename rnjuwks unemployedwks18
rename rnjusp unemployedspells18
rename rnjbsp employedspells18
rename rnjbnew employeddiffemployers18

keep pid-hhincome18 employeddiffemployers18 employedspells18 unemployedspells18
unemployedwks18 jobstatusly18 politicalparty18 dayofint18 longtermsaver18 saveregular18
monthofint18 saves18 savesamt18 ivtincome18 finsituation18 finsituationchange18
```

smoker18 cigssmoked18 hoh18 age18 region18 hsetenancy18 education18 maritalstatus18 disable18 jobstatus18 income18 hrswrk18 overtime18 commutehrs18 industry92_18 occupation90_18 sfemp18 hrswrksemp18 mental36hlth18 mental12hlth18 jobsat18 paysat18 jobsecuritysat18 jobhrssat18 subjhlth18 hlthsat18 hhincsat18 flatsat18 partnersat18 job2sat18 socialsat18 leistimesat18 leisusesat18 lifesat18 lifelysat18 religion18 neighbourchat18 meetingpeople18 spokeninlastwk18 wlkswimsprt18 livesprt18 cinema18 theatre18 eatout18 drinkout18 garden18 diycar18 eveclass18 attendgrps18 volunteer18

save "C:\BHPS Waves 16 to 18.dta", replace

*** UNDERSTANDING SOCIETY data - the 1st wave of US does not have BHPS participants - begin with wave 2 –
*** nb ensure Understanding Society individual identifier for BHPS participants matches the BHPS identifier - pid

use "C:\BHPS June 2015.dta", clear

merge 1:1 pid using "C:\xwaveid.dta"

*** WAVE B

drop pidp a_hidp a_pno a_ivfio a_ivfho b_pno b_ivfio b_ivfho c_pno c_ivfio c_ivfho d_pno d_ivfio d_ivfho _merge

merge m:1 b_hidp using "C:\UKDA-6614-stata11_se\stata11_se\b_hhresp.dta"

rename b_hid hid19
rename b_hsval hsevalue19
rename b_hscost hsecost19
rename b_mgold mortgageamt19
rename b_xpmg monthlymortloan19
rename b_nkids_dv children19
rename b_fihhmngrs_dv hhincomemonthly19
rename b_hhsize hhsize19
rename b_xphsdb hsepayprobs19

keep pid-d_hidp hid19 hsevalue19 hsecost19 mortgageamt19 monthlymortloan19 hhincomemonthly19 children19 hhsize19 hsepayprobs19
drop if pid==.
merge 1:1 pid using " C:\UKDA-6614-stata11_se\stata11_se \b_indresp.dta"

rename b_finnow finsituation19
rename b_save saves19
rename b_saved savesamt19
rename b_fiyrinvinc_dv ivtincome19
rename b_istrtdatd dayofint19
rename b_istrtdatm monthofint19
rename b_istrtdaty yearofint19
rename b_jbsic07_ industry07_19
rename b_smnow smoker19

```
rename b_smncigs cigssmoked19
rename b_jbsoc occupation90_19
rename b_jbsemp sfemp19
rename b_age_cr age19
rename b_jshrs hrswrksemp19
rename b_jbttwt commutehrs19
rename b_gor_dv regionus19
rename b_hiqual_dv education19
rename b_mastat_dv maritalstatus19
rename b_bendis3 severedisableallowance19
rename b_prfitbw incomeweekly19
rename b_prfitba incomeannual19
rename b_ff_jbstat jobstatus19
rename b_jbhrs hrswrk19
rename b_jbot overtime19
rename b_scghq1_dv mental36hlth19
rename b_scghq2_dv mental12hlth19
rename b_scsf1 subjhlthus19
rename b_sclfsat1 hlthsat19
rename b_sclfsat2 hhincsat19
rename b_sclfsat7 leistimesat19
rename b_sclfsato lifesat19
rename b_health disable19
rename b_vote4 politicalparty19
rename b_nunmpsp_dv unemployedspells19
rename b_nmpsp_dv employedspells19

keep pid-hhincomemonthly19 employedspells19 unemployedspells19 politicalparty19
yearofint19 jobstatus19 disable19 finsituation19 saves19 savesamt19 ivtincome19 dayofint19
monthofint19 industry07_19 smoker19 cigssmoked19 occupation90_19 sfemp19 age19
hrswrksemp19 commutehrs19 regionus19 education19 maritalstatus19
severedisableallowance19 incomeweekly19 incomeannual19 hrswrk19 overtime19
mental36hlth19 mental12hlth19 subjhlthus19 hlthsat19 hhincsat19 leistimesat19 lifesat19

*** mergin personality data

merge 1:1 pid using "C:\UKDA-5151-stata8\stata8\oindresp.dta"
rename optrt5a1 rude
rename optrt5a2 forgiving
rename optrt5a3 considerate
rename optrt5c1 throughjob
rename optrt5c2 lazy
rename optrt5c3 efficient
rename optrt5o1 original
rename optrt5o2 valuesart
rename optrt5o3 imaginative
rename optrt5e1 talkative
rename optrt5e2 outgoing
rename optrt5e3 reserved
rename optrt5n1 worrieslots
```

```
rename optrt5n2 nervous
rename optrt5n3 handlesstress

keep pid-ivtincome21 rude-imaginative

reshape long hid hoh employeddiffemployers employedspells unemployedspells
unemployedwks jobstatusly neighbourchat yearofint politicalparty meetingpeople
spokeninlastwk age sfemp hhincomemonthly incomeweekly subjhlthus regionus
severedisableallowance industry07_ hsepaybehind hsepaycutbacks hsepayborrow finsituation
finsituationchange hsepayprobs storecreditcards nstorecreditcards creditcardbills owesmoney
hirepurchasedebt personalloandebt creditcarddebt catalogueorderdebt socialloandebt
individualdebt overdraftdebt studentloandebt otherdebt totaldebt over500owed
over1500owed over5000owed over10000owed over100owed soledebt totalsoledebt
percenttotaldebt interestincome loans loanburden hsevalue hsecost mortgageamt
monthlymortloan longtermsaver saves savesamt saveregular ivtincome dayofint monthofint
maritalstatus religion jobstatus hrswrksemp jobsecuritysat hlthsat hhincsat flatsat partnersat
job2sat socialsat leistimesat leisusesat lifelysat wlkswimsprt livesprt cinema theatre eatout
drinkout visitfriends garden diycar eveclass attendgrps volunteer smoker cigssmoked
industry92_ incomeannual region commutehrs children hhsize disable regdisable income
hhincome hrswrk overtime industry occupation90_ hsetenancy education lifesat mental36hlth
mental12hlth jobsat paysat jobhrssat subjhlth, i(pid) j(year)

xtset pid year
```

Step 2: Generating variables

```
gsort pid year
gen hhincomemonthlyyr16=l3.hhincome*(1000/12) if year==19
replace hhincomemonthlyyr16=l4.hhincome*(1000/12) if year==20
gen lhhincomemonthly=log(hhincomemonthly)
gen lhhincomemonthlyyr16=log(hhincomemonthlyyr16)
gen changehhincome16=hhincomemonthly-hhincomemonthlyyr16 if hhincomemonthly>=0
& hhincomemonthly<20000 & hhincomemonthlyyr16>=0 & hhincomemonthlyyr16<20000
gen changelhhincome16=lhhincomemonthly-lhhincomemonthlyyr16 if hhincomemonthly>=0
& hhincomemonthly<20000 & hhincomemonthlyyr16>=0 & hhincomemonthlyyr16<20000
recode hrswrk -9=. -8=0 -2=. -1=.
gen hrswrkyr16=l3.hrswrk if year==19
gen changehrswrk16=hrswrk-hrswrkyr16

replace lifesat=. if lifesat<1
replace mental36hlth=. if mental36hlth<0
su lifesat mental36hlth
***     Variable |       Obs        Mean    Std. Dev.       Min        Max
*** -------------+---------------------------------------------------------
***      lifesat |     70051    5.191532    1.364876          1          7
*** mental36hlth |     67803    11.22598    5.579457          0         36

egen stdlifesat=std(lifesat)
egen stdmental36hlth=std(mental36hlth)
```

```
gen region1=regionus
recode region1 1=1 2=1 3=1 4=2 5=2 6=2 7=3 8=4 9=4 10=5 11=6 12=7
label define region1 1 "The North" 2 "Midland and East" 3 "London" 4 "The South" 5
"Wales" 6 "Scotland" 7 "Northern Ireland"
label values region1 region1

gen stdlifesatyr16=.
replace stdlifesatyr16=l3.stdlifesat if year==19
gen stdmental36hlthyr16=.
replace stdmental36hlthyr16=l3.stdmental36hlth if year==19

recode age -9=.
su l1.age if age>0 & year==19
gen centage=age-46.75323

recode education .=99
recode region1 .=99
recode jobstatus .=99

recode sex 99=.

gen educationyr16=l3.education if year==19

gen ageyr16=l3.age if year==19

gen region1yr16=l3.region1 if year==19

gen jobstatusyr16=.
replace jobstatusyr16=l3.jobstatus if year==19
gen lhhsizeyr16=.
replace lhhsizeyr16=l3.lhhsize if year==19

*** unemployment transitions - 2006/7 to 2009/10
*** some unemployment
gen someunemploymentyr16=.
recode someunemploymentyr16 .=1 if unemployedspells>0 & unemployedspells!=. &
year==19
recode someunemploymentyr16 .=1 if l1.unemployedspells>0 & l1.unemployedspells!=. &
year==19
recode someunemploymentyr16 .=1 if l2.unemployedspells>0 & l2.unemployedspells!=. &
year==19
recode someunemploymentyr16 .=0 if unemployedspells!=. & year==19
recode someunemploymentyr16 .=0 if l1.unemployedspells!=. & year==19
recode someunemploymentyr16 .=0 if l2.unemployedspells!=. & year==19
recode someunemploymentyr16 .=1 if unemployedspells>0 & unemployedspells!=. &
year==20
recode someunemploymentyr16 .=1 if l1.unemployedspells>0 & l1.unemployedspells!=. &
year==20
recode someunemploymentyr16 .=1 if l2.unemployedspells>0 & l2.unemployedspells!=. &
year==20
```

```
recode someunemploymentyr16 .=1 if l3.unemployedspells>0 & l3.unemployedspells!=. &
year==20
recode someunemploymentyr16 .=0 if unemployedspells!=. & year==20
recode someunemploymentyr16 .=0 if l1.unemployedspells!=. & year==20
recode someunemploymentyr16 .=0 if l2.unemployedspells!=. & year==20
recode someunemploymentyr16 .=0 if l3.unemployedspells!=. & year==20
```

Step 3: Descriptive statistics and multiple imputation

```
*** SWB differences - Table 1
su l3.lifesat if year==19 & lifesat!=. & l3.lifesat!=., de
su l2.lifesat if year==19 & lifesat!=. & l1.lifesat!=. & l2.lifesat!=., de
su l1.lifesat if year==19 & lifesat!=. & l1.lifesat!=. & l3.lifesat!=., de
su lifesat if year==19 & lifesat!=. & l3.lifesat!=., de

count if year==19 & l3.lifesat==1 & lifesat!=. & l3.lifesat!=.
count if year==19 & l3.lifesat==2 & lifesat!=. & l3.lifesat!=.
count if year==19 & l3.lifesat==3 & lifesat!=. & l3.lifesat!=.
count if year==19 & l3.lifesat==4 & lifesat!=. & l3.lifesat!=.
count if year==19 & l3.lifesat==5 & lifesat!=. & l3.lifesat!=.
count if year==19 & l3.lifesat==6 & lifesat!=. & l3.lifesat!=.
count if year==19 & l3.lifesat==7 & lifesat!=. & l3.lifesat!=.

count if year==19 & lifesat==1 & lifesat!=. & l3.lifesat!=.
count if year==19 & lifesat==2 & lifesat!=. & l3.lifesat!=.
count if year==19 & lifesat==3 & lifesat!=. & l3.lifesat!=.
count if year==19 & lifesat==4 & lifesat!=. & l3.lifesat!=.
count if year==19 & lifesat==5 & lifesat!=. & l3.lifesat!=.
count if year==19 & lifesat==6 & lifesat!=. & l3.lifesat!=.
count if year==19 & lifesat==7 & lifesat!=. & l3.lifesat!=.

gen lifesatyr16=l3.lifesat
ttest lifesatyr16 = lifesat if year==19 & lifesat!=. & l3.lifesat!=.

gen lifesatyr16=l3.lifesat
keep if year==19 & stdlifesat!=. & stdlifesatyr16!=.
gen hhsizeyr16=exp(lhhsizeyr16)
su lifesat lifesatyr16
su ageyr16 i.sex hhincomemonthlyyr16 hrswrkyr16 hhsizeyr16 changehrswrk
someunemploymentyr16 changehhincome16
su 1.educationyr16
su b2.educationyr16
su i.region1yr16
su b2.region1yr16
su i.jobstatusyr16
su b2.jobstatusyr16
gen ageyr16sq=ageyr16^2
ttest lifesat = lifesatyr16
gen changelifesat=lifesat-lifesatyr16
bysort sex: ttest changelifesat=0
```

```
bysort educationyr16: ttest changelifesat=0
bysort region1yr16: ttest changelifesat=0
bysort someunemploymentyr16: ttest changelifesat=0
bysort jobstatusyr16: ttest changelifesat=0

*** Multiple Imputation
mi set mlong
*** summary statistics
su ageyr16 hrswrkyr16 changehrswrk changelhhincome16 someunemploymentyr16
lhhsizeyr16 lhhincomemonthlyyr16 educationyr16 region1yr16 jobstatusyr16 sex
mi register imputed ageyr16 ageyr16sq hrswrkyr16 changehrswrk changelhhincome16
someunemploymentyr16 lhhincomemonthlyyr16
set seed 12345
mi impute chained (pmm, knn(3)) ageyr16 (pmm, knn(3)) ageyr16sq (pmm, knn(3))
hrswrkyr16 (pmm, knn(3)) changehrswrk (pmm, knn(3)) changelhhincome16 (logit)
someunemploymentyr16 (pmm, knn(3)) lhhincomemonthlyyr16 = b1.sex b9.educationyr16
b7.region1yr16 b2.jobstatusyr16 lhhsizeyr16, add(5) augment noisily
```

Step 4: Regressions

```
*** first centre variables
cd c:\Temp
mi xeq 0: su ageyr16 ageyr16sq lhhincomemonthlyyr16 lhhsizeyr16 changelhhincome16
changehrswrk hrswrkyr16
mi xeq: gen centageyr16=ageyr16-46.39654
mi xeq: gen centageyr16sq=ageyr16sq-2453.17
mi xeq: gen centlhhincomemonthlyyr16=lhhincomemonthlyyr16-7.732137
mi xeq: gen centlhhsizeyr16=lhhsizeyr16-.9311076
mi xeq: gen centchangelhhincome16=changelhhincome16-.1811137
mi xeq: gen centchangehrswrk=changehrswrk+0.8749595
mi xeq: gen centhrswrkyr16=hrswrkyr16-17.81868

*** summary statistics for SWB variables
mi xeq: su lifesat
*** from 2006 to 2010 -need to redo mi with correct age categories and then re-estimate
*** empty model
mi estimate: reg stdlifesat stdlifesatyr16 if year==19, cluster(pid)

*** predicting recession consequences - Table 2
*** INCOME
mi estimate: reg changelhhincome16 centageyr16 centageyr16sq i.sex b9.educationyr16
b3.region1yr16 if year==19 & age>0, cluster(pid)
mibeta changelhhincome16 ageyr16 ageyr16sq i.sex b9.educationyr16 b3.region1yr16 if
year==19 & ageyr16>0, cluster(pid)
*** UNEMPLOYMENT
mi estimate: probit someunemploymentyr16 centageyr16 centageyr16sq i.sex
b9.educationyr16 b3.region1yr16 if year==19 & age>0, cluster(pid)
mibeta someunemploymentyr16 ageyr16 ageyr16sq  i.sex b9.educationyr16 b3.region1yr16 if
year==19 & age>0, cluster(pid)
*** LOSS of WORK HOURS
```

```
mi estimate: reg changehrswrk centageyr16 centageyr16sq i.sex b9.educationyr16
b3.region1yr16 if year==19 & age>0, cluster(pid)
mibeta changehrswrk ageyr16 ageyr16sq i.sex b9.educationyr16 b3.region1yr16 if year==19
& age>0, cluster(pid)

*** Recession well-being effects - Table 3
*** t-1 and t no controls
mi estimate: reg stdlifesat stdlifesatyr16 if year==19 & ageyr16>0, cluster(pid)
mibeta stdlifesat stdlifesatyr16 if year==19 & ageyr16>0, cluster(pid)
*** demograhpic variables
mi estimate: reg stdlifesat stdlifesatyr16 centageyr16 centageyr16sq i.sex b9.educationyr16
b7.region1yr16 if year==19 & ageyr16>0, cluster(pid)
mibeta stdlifesat stdlifesatyr16 ageyr16 ageyr16sq i.sex b9.educationyr16 b3.region1yr16 if
year==19 & ageyr16>0, cluster(pid)
*** t-1 and t economic and psych variables - no loss effect for income
mi estimate: reg stdlifesat stdlifesatyr16 centageyr16 centageyr16sq i.sex b9.educationyr16
b7.region1yr16 b2.jobstatusyr16 centlhhincomemonthlyyr16 hrswrkyr16 centlhhsizeyr16
someunemploymentyr16 changehrswrk centchangelhhincome16, cluster(pid)
mimrgns
mimrgns, at(someunemploymentyr16=(0 1))
mi xeq 0: count if someunemploymentyr16==1
mimrgns, at(jobstatusyr16=8)
mi xeq 0: count if jobstatusyr16==8
mimrgns, at(jobstatusyr16=2 educationyr16=1 centlhhincomemonthlyyr16=1)
mi xeq 0: count if jobstatusyr16==2 & educationyr16==1 & centlhhincomemonthlyyr16>=1
mimrgns, at(educationyr16=9 region1yr16=3)
mi xeq 0: count if educationyr16==9 & region1yr16==3

mibeta stdlifesat stdlifesatyr16 ageyr16 ageyr16sq i.sex b9.educationyr16 b3.region1yr16
b2.jobstatusyr16 centlhhincomemonthlyyr16 hrswrkyr16 centlhhsizeyr16
someunemploymentyr16 changehrswrk centchangelhhincome16 if year==19 & ageyr16>0,
cluster(pid)

*** non-imputed regresssions
*** INCOME

use "C:\Users\Laptop\Documents\Research projects (current)\Political, Policy, and Well-
Being\Great Recession data - before MI V1a.dta",clear
gen lifesatyr16=l3.lifesat
keep if year==19 & stdlifesat!=. & stdlifesatyr16!=.
gen hhsizeyr16=exp(lhhsizeyr16)
gen ageyr16sq=ageyr16^2

reg changelhhincome16 ageyr16 ageyr16sq i.sex b9.educationyr16 b3.region1yr16 if
year==19 & age>0, cluster(pid)
*** UNEMPLOYMENT
reg someunemploymentyr16 ageyr16 ageyr16sq i.sex b9.educationyr16 b3.region1yr16 if
year==19 & age>0, cluster(pid)
*** LOSS of WORK HOURS
```

reg changehrswrk ageyr16 ageyr16sq i.sex b9.educationyr16 b3.region1yr16 if year==19 & age>0, cluster(pid)

*** Recession well-being effects - Table 3
*** t-1 and t no controls
reg stdlifesat stdlifesatyr16 if year==19 & ageyr16>0, cluster(pid)
*** demograhpic variables
reg stdlifesat stdlifesatyr16 ageyr16 ageyr16sq i.sex b9.educationyr16 b7.region1yr16 if year==19 & ageyr16>0, cluster(pid)
*** t-1 and t economic and psych variables - no loss effect for income
reg stdlifesat stdlifesatyr16 ageyr16 ageyr16sq i.sex b9.educationyr16 b7.region1yr16 b2.jobstatusyr16 lhhincomemonthlyyr16 hrswrkyr16 lhhsizeyr16 someunemploymentyr16 changehrswrk changelhhincome16 if year==19 & ageyr16>0, cluster(pid)
